# The Role of Early Visual Experience in Cross‐Signal Dependency Detection

**DOI:** 10.1111/desc.70204

**Published:** 2026-05-05

**Authors:** Priti Gupta, Lukas Vogelsang, Marin Vogelsang, Neil Khemani, Manvi Jain, Naviya Lall, Dhun Verma, Chetan Ralekar, Suma Ganesh, Pawan Sinha

**Affiliations:** ^1^ Boston Children's Hospital Harvard Medical School Boston Massachusetts USA; ^2^ Project Prakash, Dr Shroff's Charity Eye Hospital New Delhi Delhi India; ^3^ Cognitive Science Program Dayalbagh Educational Institute Agra India; ^4^ Department of Brain and Cognitive Sciences Massachusetts Institute of Technology Cambridge Massachusetts USA; ^5^ Department of Computer Science Stanford University California USA; ^6^ Department of Humanities and Social Sciences Indian Institute of Technology Delhi Delhi India; ^7^ Mehta Family School of Data Science and Artificial Intelligence Indian Institute of Technology Guwahati Assam India; ^8^ Department of Pediatric Ophthalmology Dr Shroff's Charity Eye Hospital New Delhi Delhi India

**Keywords:** congenital blindness, correlation detection, late sight onset, temporal coincidences, visual development

## Abstract

Temporal co‐occurrence of two sensory signals is a powerful indicator of their potential association. Detection of such coincidences is believed to be a key contributor to perceptual organization and a primary objective of cortical computations. Here, we investigate whether the development of this ability requires early sensory experience. We examined the performance of early‐blind children who surgically gained sight as adolescents. Our data revealed three key results. First, we found that while not fully reaching the level of normally sighted controls, late‐sighted patients examined several years after surgery were able to robustly detect signal dependencies with markedly above‐chance accuracy at all but the weakest strengths tested. Second, for both groups, performance levels were comparable for intra‐ and inter‐modal stimuli, revealing significant plasticity for establishing cross‐modal linkages late in childhood. Finally, comparison with a separate group of late‐sighted patients tracked longitudinally from preoperative to one‐month postoperative status suggests that this ability may follow a protracted developmental time course after sight surgery. These results help characterize the development of a foundational process for detecting relationships between environmental entities and point to the resilience of acquiring this important skill to early‐onset, prolonged visual deprivation.

**Summary:**

- We tested the ability of congenitally blind children who gained sight late in childhood to detect repeated temporal co‐occurrence of sensory signals.
- We found that while not reaching the full normally sighted level, late‐sighted patients are able to detect signal dependencies with markedly above‐chance accuracy.
- This ability may follow a protracted developmental time course after sight‐restoring surgery.
- The availability of neural plasticity into late childhood has important implications for the rehabilitation prospects of children with congenital blindness.

## 1 Introduction

The eminent neuroscientist, Horace Barlow (Burr and Laughlin 2020), proposed that a key purpose of cortical computations is to detect “suspicious coincidences” in incoming signals (Barlow 1994). When two signals co‐occur more often than would be the case if they were truly independent (i.e., when the joint probability of two events, A and B, exceeds the product of individual base rates; *P*(*A*∩*B*) > *P*(*A*)*P*(*B*)), they are said to exhibit suspicious coincidences. If detected, these coincidences provide evidence that the observed sensory signals potentially arise from a common cause (Blake and Lee 2005; Parise et al. 2012) and hence ought to be integrated, even when other cues, such as their spatial locations, suggest otherwise (Parise et al. 2012; Parise and Ernst 2016).

Given its functional importance, the study of cross‐modal temporal relationship perception has attracted significant research attention. Most investigations have focused on two processes that are required for, but distinct from, suspicious coincidence detection: Simultaneity perception, which describes the ability to discern whether or not two discrete sensory inputs transpired together in time (Mitrani et al. 1986; Spence et al. 2001; Zampini et al. 2005; Roseboom et al. 2009), and synchrony perception, which builds on the capacity to detect whether two sensory streams are precisely aligned in time (Dixon and Spitz 1980; Vatakis and Spence 2006; Vroomen and Keetels 2010; Maier et al. 2011). Although an important ability in its own right, simultaneity detection cannot be extrapolated to predict suspicious coincidence detection performance with extended sequences. This is because the latter task requires temporal integration across multiple event instances in the input streams rather than just one isolated pair (since whether or not a co‐occurrence is “suspicious” depends on an accumulation of evidence to estimate base rates and joint probabilities). Synchrony detection does require temporal integration but, in presenting perfect correspondence between events in the two signals, is a very specific instance of the more general dependency detection task, in which the signal linkage may be imperfect (i.e., not all events may be in correspondence across the two signals). We will henceforth adopt the teleologically richer phrasing and refer to this as cross‐signal dependency detection.

Several important questions about the basic characterization of this capacity remain open. The key question we focus on in the present study concerns the role of early visual experience in the development of this important ability, which, together with other time‐based proficiencies, may play a key role in helping to organize the complex sensorium (Sinha et al. 2025). Specifically, we ask whether early exposure to temporally dependent and independent sensory streams is necessary for humans to be able to detect cross‐signal dependencies later in life. To address this issue, we have had an opportunity to examine whether a unique group of visual observers we have been working with—patients treated for congenital blindness late in childhood—are able to acquire this proficiency despite the prolonged period of deprivation they suffered.

Past work with late‐sighted children has provided evidence indicating the criticality of early experience for key dimensions of spatial visual processing. This includes resolution acuity, vernier acuity, and pattern vision (Ganesh et al. 2014; Vogelsang et al. 2025; Kalia et al. 2014; Lewis and Maurer 2005), shape recognition (McKyton et al. 2015), robustness to degradation (Vogelsang et al. 2024), as well as facial identification and attractiveness perception (Le Grand et al. 2004; Vogelsang et al. 2018; Gupta et al. 2023). In contrast to spatial visual processing, the pattern of results in the temporal domain appears to be more encouraging. Late‐sighted children are able to perceive motion (albeit with elevated global motion coherence thresholds; Ellemberg et al. 2002) and use this ability to parse dynamic imagery into distinct objects (Ostrovsky et al. 2009). Additionally, their temporal contrast sensitivity is notably robust against visual deprivation, and much more so than its spatial counterpart (Ellemberg et al. 1999; Ye et al. 2021). Late‐sighted children are also able to perceive shapes presented anorthoscopically by linking oriented segments across a continuous temporal sequence (Orlov et al. 2021).

Notwithstanding these results, we cannot directly extrapolate the children's proficiency on the dependency detection task, which requires the detection of simultaneity and temporal integration across several discontinuous instances of co‐occurrence. Adding to the task's complexity are the facts that the co‐occurrences are probabilistic and can occur between entities within the same sensory modality or across different modalities. Given these unique task characteristics, it is difficult to predict a‐priori the late‐sighted children's capacity.

In fact, even as basic temporal sensitivity may be relatively preserved, there are reasons to expect that severe early spatial degradation would impact performance on a temporal dependency detection task. The spatial blur characteristic of dense congenital cataracts limits the ability to resolve nearby, distinct visual entities, which are precisely those that are most likely to exhibit meaningful temporal covariation in natural environments. With fewer opportunities to perceive individuated elements that covary over time, it is plausible that the development of mechanisms for learning such temporal relationships could be compromised. This, coupled with the ecological significance of the dependency detection task, motivates examining it in the late‐sighted population.

With this approach, we seek to address four research questions. First, what is the impact of early‐onset visual deprivation on the ability to detect temporal sensory dependencies of varying strengths? Second, are postoperative improvements, if any, immediate, or do they follow a gradual development? Third, are late‐sighted patients and normally sighted controls more accurate at detecting intra‐modal dependencies in the visual system or inter‐modal ones between vision and audition? Fourth, and finally, how much information, relative to a theoretically determined criterion, do observers across groups require to reliably detect dependency between two signals?

## 2 Methods

### 2.1 Patient Groups

We recruited two nonoverlapping groups of late‐sighted patients. Our first group comprised 15 long‐term follow‐up patients (5 females; mean age: 17.5 years) who had undergone surgical intervention for congenital blindness at ages ranging from 8 to 16 years (see Table S1 for details). They were tested three or more years after their surgeries. Our second group included 12 longitudinally‐tracked patients (9 females; mean age: 9.8 years; detailed patient data in Table S2). For both groups, patients were identified through our pediatric ophthalmic screening program conducted in rural areas of India. The assessment of congenitality of deprivation relied on multiple factors, including parental reports, eyeball and cataract morphology, as well as the presence of nystagmus, which is a known consequence of profound, early visual impairment (Tusa et al. 1991). Details about the surgical intervention and procedures can be found in the Supplementary Methods.

### 2.2 Control Group

Our control group comprised 21 participants (21 females; mean age: 18.9 years; Table S3) with normal or corrected‐to‐normal vision and no history of any neurological conditions. Informed consent was obtained from all participants. To roughly match the control participants’ visual acuity with that of the patients, we used on‐screen blurring corresponding to 20/400 vision (for 10 participants) and 20/500 vision (for 11 participants). Note that this was just a precaution; the experiment was designed with large, high‐contrast stimuli to reduce the reliance on high visual acuity. As elaborated on in the Discussion section, acuity differences at this level did not meaningfully affect performance (see Figure S1A; Supplementary Methods).

It is important to note that recruitment of participants for studies involving late‐sighted individuals presents several practical challenges. In addition to the difficulties inherent in identifying and testing this rare population, it is also challenging to recruit appropriately matched control participants. Given the logistical difficulty of bringing in external school‐aged controls from comparable socioeconomic backgrounds, in the present study, we recruited control participants from on‐site nursing student cohorts at the hospital, whose socioeconomic background broadly matches that of the patient families. Given that nursing programs in India are overwhelmingly female, the control group in the present study comprised only female participants. While we are not aware of any sex‐linked differences in temporal dependency detection, we note this sampling characteristic for completeness and transparency.

### 2.3 Demographic Information

All children in our experimental and control groups were ethnically Indian and come from impoverished socioeconomic backgrounds from remote rural parts of India. Both groups' economic status is indicated by their household income, which was under $100 per month, qualifying their categorization as being far below the “extreme poverty” group as defined by UN guidelines (corresponding to less than $2.15 per person per day at 2017 purchasing power. Note that $100 in our sample corresponds to the income of the entire household, which typically has 5 or more members, translating to a daily amount of about $0.67 per person). None of the families had maternal/paternal education beyond high school, and typically much less (mothers were generally not educated beyond grade 5 of primary school). An indication of the experimental group's poverty is also evident in the fact that the screening by our team was their first contact with the medical community. The families' financial resources were so limited as to preclude even modest medical care. Ages and genders of individual participants are provided in Tables S1–S3.

### 2.4 Stimuli

**FIGURE 1:**
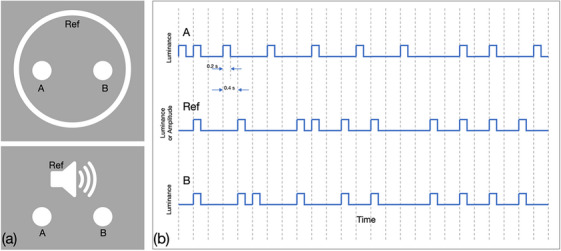
**A**. Design of the stimuli in the intra‐ and inter‐modal conditions (upper and lower panels, respectively). The intramodal condition comprised three visual elements: two discs and a surrounding annulus. The participants’ task was to determine which of the two discs was more correlated with the reference annulus over the course of a 20‐second sequence. The inter‐modal condition involved replacing the visual annulus with a concurrent audio track of beeps and silences. **B**. Schematic illustration of the stimulus sequences (the actual sequences used in the study were 50 elements long). In the example depicted here: All sequences have a base rate of 0.4; *P*(A, Ref) = 4/25 = *P*(A)*P*(Ref); *P*(B, Ref) = 9/25 > *P*(B)P(Ref).

Our experimental paradigm comprised intra‐modal (visual‐visual) and inter‐modal (audio‐visual) conditions. In the intra‐modal condition (see Figure 1A), participants were presented with two disks (“A” and “B”) surrounded by an annulus (“Ref” for “reference”). They were instructed to determine which of the two disks was flashing most in unison with the circumscribing annulus. The luminance of the visual elements was modulated in accordance with pre‐generated binary sequences (see Figure 1B for an exemplar sequence). In the inter‐modal condition, participants had to identify which of the two disks was flashing most congruently with a concurrent audio track of beeps and silences. All sequences were generated prior to the experiment.

The construction of the binary sequences is schematized in Figure 1B. All three sequences were restricted to be “active” (i.e., flashing or beeping) for a total of 20 out of the 50 sequence elements (i.e., *P*(*A*) = *P*(*B*) = *P*(Ref) = 0.4). A fully‐independent stream (in the illustration, “A”) was generated by enforcing that the probability of elements in streams “A” and “Ref” being active simultaneously was equivalent to the product of the probabilities of them being active individually (*P*(*A*)*P(Ref) = 40% * 40% = 16%, i.e., flashing/beeping together for 8 of the 50 sequence elements). The dependent stream (in the illustration, “B”) was generated by ensuring that streams “B” and “Ref” were simultaneously active more often than if they were fully independent (i.e., for more than 16% of the stimulus elements). In the experiment, we examined the following joint probabilities: 20%, 28%, 32%, 36%, and 40%, ranging from only marginally dependent (20%) to fully‐dependent (40%). For each of the five different strengths of dependency, five different sequences were constructed (the same sequences were used for intermodal and intramodal conditions). The order of sequence presentation was randomized across participants.

Each sequence element was presented for 0.4 s (if the sequence element was an active one, the flash/beep lasted for 0.2 s of the 0.4 second duration). The presentation of the entire 50‐element sequence, therefore, took 20 s. Participants’ primary task, following the presentation of the entire stimulus sequence, was to report which of the two streams, “A” or “B,” was more correlated with the reference stream.

Participants were instructed to report their decision as soon as they felt confident about their choice, and to respond again at the end of the trial. The index of the sequence element at which the initial decision was made was used to estimate how much evidence participants required for a decision, and to compare this value with theoretical decision thresholds derived from a statistical criterion (see Supplementary Methods).

In the long‐term follow‐up cohort, the option to report an early decision was introduced after nine participants had already completed testing. Consequently, six of fifteen patients in this group provided both an initial and a final response, whereas the other nine participants provided only a final response. All participants in the other groups provided both responses. As elaborated on in the Discussion section, the requirement to provide an initial response in addition to a final response did not affect performance (see Figure S1B; Supplementary Methods).

### 2.5 Experimental Procedure

Prior to the experiment, participants were presented with visualized instruction slides, which were also explained to them by the experimenter. Whether a given participant was first presented with the intermodal or the intramodal condition was determined randomly. For each condition, participants were shown 5 high‐dependency practice sequences (joint probability = 0.4) to familiarize them with the task and to allow for clarification of any questions before beginning the main experiment. These practice sequences were not trivially easy (e.g., catch trials) but corresponded to the easiest dependency level used in the experiment and were intended solely as instructional demonstrations and were not used as performance‐based inclusion or exclusion criteria. Participants subsequently completed the main experiment for that condition (comprising 25 trials; 5 per probability level, with the presentation order randomly shuffled across participants).

## 3 Results

### 3.1 Performance of Late‐Sighted Patients Several Years After Surgery

**FIGURE 2:**
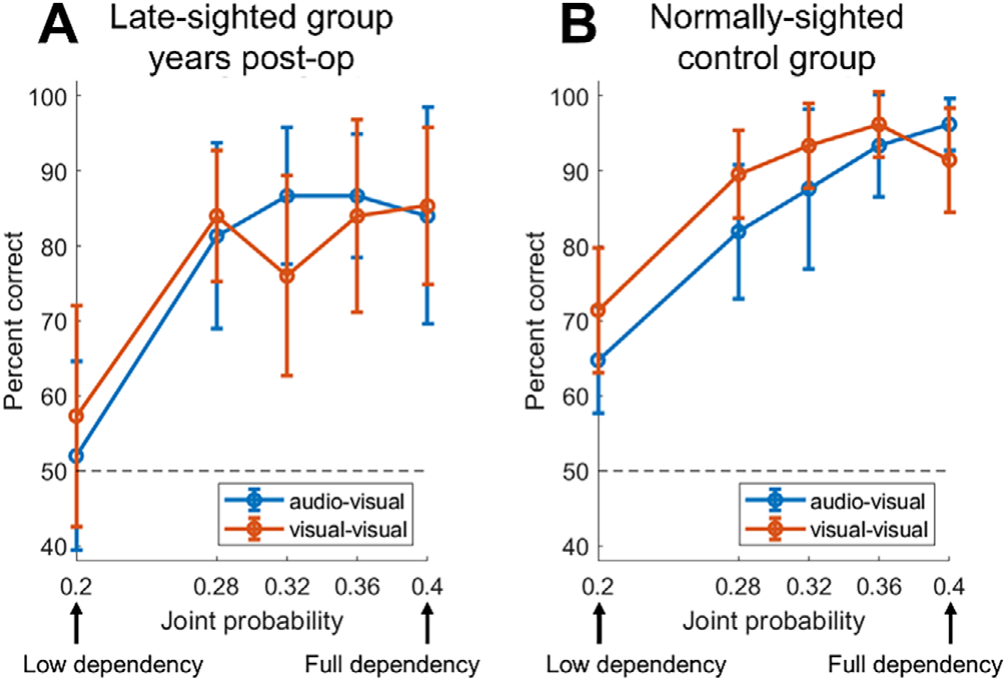
**A and B**. Performance (in percent correct) of the late‐sighted (*n* = 15) and control (*n* = 21) groups, as a function of probabilistic dependency strength (0.20 = only marginally dependent; 0.4 = fully‐dependent) and modality (audio‐visual vs. visual‐visual). Error bars depict 95% confidence intervals.

Figure 2 depicts the mean performance of late‐sighted patients several years post‐surgery and normally sighted controls as a function of modality (audio‐visual vs. visual‐visual) as well as probabilistic dependency strength, ranging from only marginally dependent (at 0.2; full independence would be at 0.16) to fully‐dependent (at 0.4).

First, we observe that the performance of the patient group on all except the weakest probabilistic dependency strengths is significantly above chance (significant Bonferroni‐corrected Wilcoxon signed rank tests comparing observed and chance‐level performance; see Table S4). Control participants exhibit significant above‐chance performance at all levels of dependency tested (Table S5).

To quantify how the performance profiles of late‐sighted individuals compare with those of blur‐matched normally sighted controls, we carried out a 3‐way mixed ANOVA on performance scores (see Table 1). This analysis revealed a significant main effect of probabilistic dependency strength (*F* = 31.005, *p* < 0.001) with a very large effect size (η_***p***_ **^2^** = 0.477) as well as a significant group difference (*F* = 4.655, *p* = 0.038) with a medium effect size (η_***p***_ **^2^** = 0.120). Thus, while exhibiting markedly above‐chance dependency detection capabilities, the performance of the late‐sighted patient group (Mean performance: 77.7%) is still slightly, but statistically significantly, lower than that of normally sighted controls (Mean performance: 86.6%). However, there was no significant effect of modality (*F* = 0.440, *p* = 0.512) or a <group × modality> interaction effect (*F* = 1.080, *p* = 0.306), indicating that performance on the intermodal and intramodal condition is comparable for both groups.

**TABLE 1:** Tests of within‐subject effects and between‐subject effects of 3‐way mixed ANOVA on performance scores, carried out in SPSS, reporting type III sum of squares, degrees of freedom, mean squares, *F* value, *p*‐value (bold if P < 0.05) and partial eta squared, each based on Greenhouse‐Geisser results as sphericity was not assumed.

Variable	SS	Df	MS	*F*	*P*	η_*p*_^2^
**Within‐subject effects**:						
Modality	0.017	1	0.017	0.440	0.512	0.013
Modality * group	0.043	1	0.043	1.080	0.306	0.031
Probability	3.936	2.995	1.314	31.005	**<0.001**	0.477
Probability * group	0.096	2.995	0.032	0.760	0.519	0.022
Modality * probability	0.108	2.775	0.039	1.408	0.247	0.040
Modality * probability * group	0.116	2.775	0.042	1.513	0.219	0.043
**Between‐subject effects**:						
Group	0.683	1	0.683	4.655	**0.038**	0.120

In summary, although their performance is statistically lower than that of controls, late‐sighted patients are capable of acquiring a remarkably above‐chance ability to detect sensory dependencies despite the early‐onset extended visual deprivation they have suffered.

### 3.2 Performance of the Late‐Sighted Immediately Following Surgery

In light of the relatively high performance levels exhibited by the late‐sighted group several years after surgery, we next examined whether this skill is available immediately postoperatively or whether it develops gradually thereafter. To this end, we longitudinally tracked the status of 12 congenitally blind children across four time points, from preoperatively (“pre‐op”) to 1‐month postoperatively (“post‐op 3”). Note that these children do not overlap with individuals from the long‐term follow‐up group.

**FIGURE 3:**
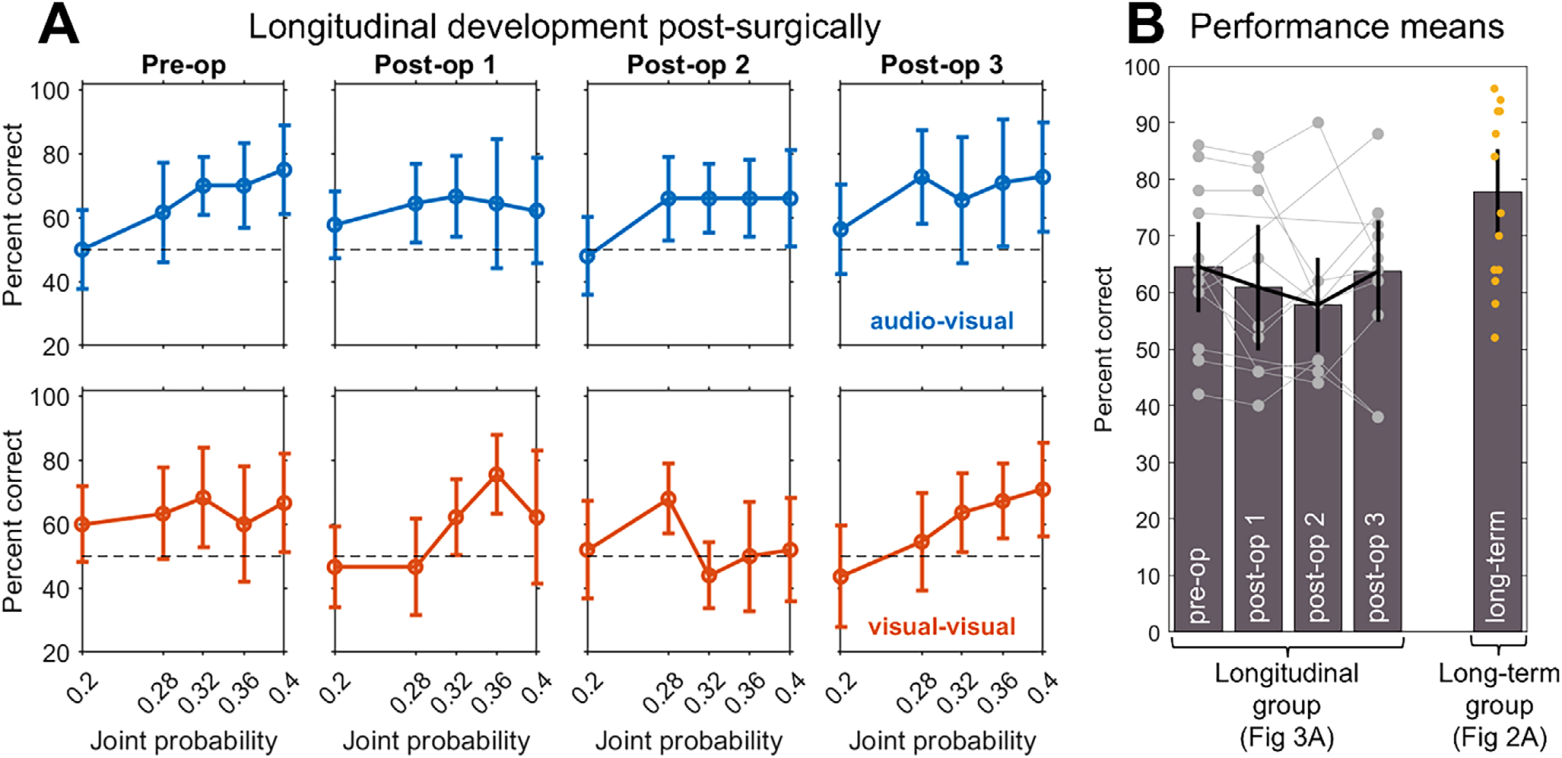
**A**. Performance (in percent correct) of the longitudinally‐tracked late‐sighted group as a function of probabilistic dependency strength and modality. The four time points correspond to pre‐surgical status (n = 12), and approximately 1 week (*n* = 9), 2 weeks (*n* = 10), and 1 month (*n* = 11) post‐surgery. **B**. Performance averaged across probabilistic dependency strength and modality, for each of the four longitudinal time points, and compared to those of the (nonoverlapping) group of long‐term follow‐up patients. Error bars in panels A and B depict 95% confidence intervals.

Across the four longitudinal time points, no clear improvements can be observed (see Figure 3A). Moreover, comparing performance means of the longitudinally assessed group 1‐month post surgically with those of the long‐term follow‐up group (see Figure 3B), we observe significant differences between the two (*t*(24) = 2.328; *p* = 0.0287 in two‐tailed, two‐sample *t*‐test of mean performance). Thus, it is possible that the post surgical improvement is not immediate but follows a protracted progression, well beyond one month. In light of the challenges of cross‐sectional comparisons, we discuss this interpretation at greater length in the Discussion section.

As visible in Figure 3A, it is important to note that mean performance in the longitudinal cohort was modestly above chance already preoperatively in both modalities. This finding likely reflects the patients’ residual vision in combination with the low spatial acuity demands of the large, high‐contrast stimuli used in the experiment. However, performance remained markedly below that exhibited by the group of children who have had several years of post surgical visual experience.

### 3.3 Analysis of the Time Point at Which the First Decisions Were Made

**FIGURE 4:**
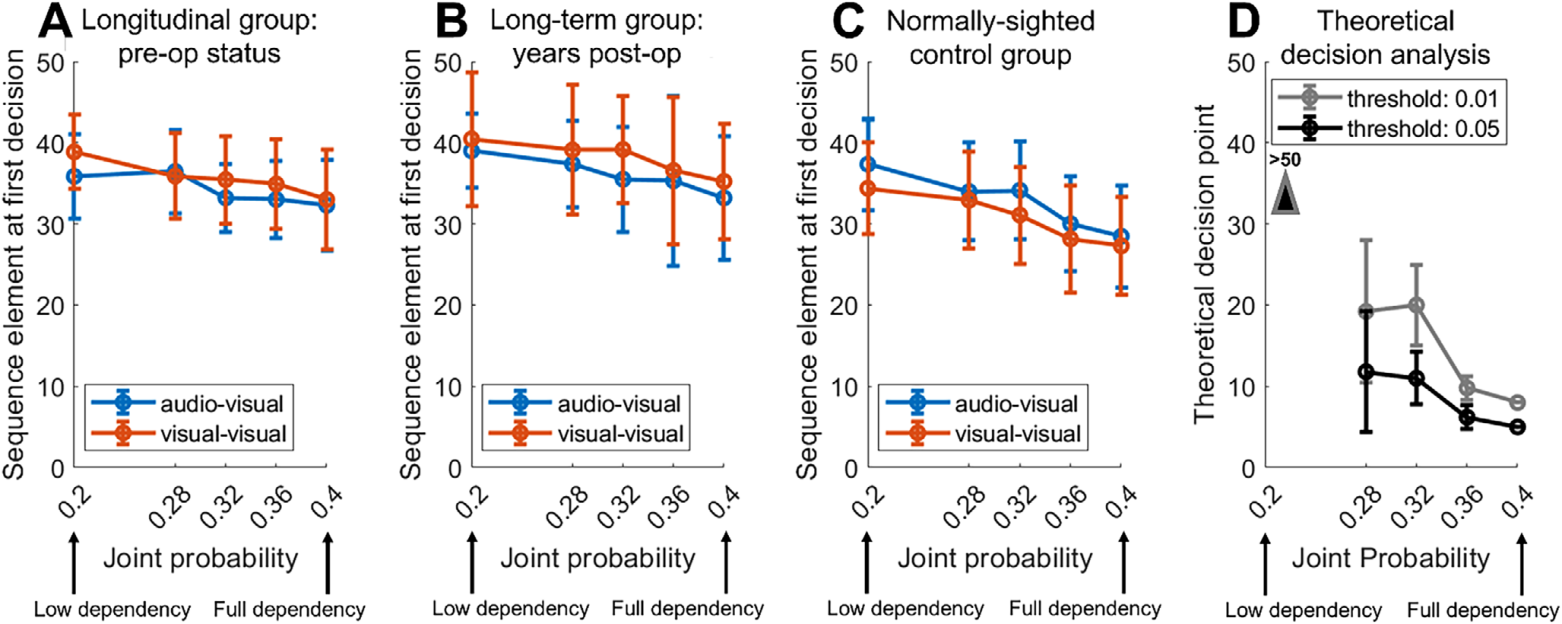
**A–C**. Sequence element at which the first decision was made, depicted separately for the longitudinal group (*n* = 12) at the preoperative time point, the (nonoverlapping) late‐sighted group examined several years post surgically (*n* = 6), as well as controls (*n* = 21), as a function of probabilistic dependency strength (0.16 = fully‐independent; 0.4 = fully‐dependent) and modality (audio‐visual vs. visual‐visual). **D**. Theoretical decision points deriving from applying the *p* < 0.05 and *p* < 0.01 criteria to the probability of observing m or more sequence element matches between a given stream and the reference stream in an n‐long sequence.

Complementing the examination of normally‐ and late‐sighted individuals’ decision accuracy, we analyzed the time at which their first decisions were made. This analysis was included to examine how much evidence observers required before committing to a choice and to assess whether participants differed in their decision thresholds across groups. Figure 4A‐C depict the means and confidence intervals of the first decision points (ranging from 1 to 50 sequence elements shown) for three independent groups: the longitudinal group (*n* = 12), a subset of the late‐sighted group several years post‐surgery (n = 6) and the control (*n* = 21) group. This analysis was conducted as a function of modality and probabilistic dependency strength. For the longitudinal cohort, the preoperative time point is depicted to show the patients’ visual status is at the most compromised level. Patterns at later time points post‐surgery were qualitatively similar (see Figure S2).

As quantified by a 3‐way ANOVA on such decision times, while there is a significant main effect of probabilistic dependency strength (*F* = 13.116, *p* < 0.001), there is no significant group difference (*F* = 0.689, *p* = 0.508), <group × modality> interaction (*F* = 1.482, *p* = 0.241), or <group × dependency strength> interaction (*F* = 0.855, *p* = 0.526) (see Table 2). Thus, the observed decision points do not differ significantly across the three groups.

**TABLE 2:** Tests of within‐subject effects and between‐subject effects of 3‐way mixed ANOVA on first response time, carried out in SPSS, reporting type III sum of squares, degrees of freedom, mean squares, *F* value, *p*‐value (bold if P < 0.05) and partial eta squared, each based on Greenhouse‐Geisser results as sphericity was not assumed.

Variable	SS	Df	MS	*F*	*P*	η_*p*_^2^
**Within‐subject effects**:						
Modality	18.199	1	18.199	0.163	0.689	0.005
Modality * group	331.052	2	165.526	1.482	0.241	0.076
Probability	1384.592	2.840	487.613	13.116	**<0.001**	0.267
Probability * group	180.483	5.679	31.780	0.855	0.526	0.045
Modality * probability	6.466	3.515	1.840	0.078	0.982	0.002
Modality * probability * group	94.407	7.030	13.430	0.573	0.778	0.031
**Between‐subject effects**:						
Group	1628.996	2	814.498	0.689	0.508	0.037

We also examined how the empirically observed decision points would compare to the amount of information theoretically required by a hypothetical observer to arrive at a decision with a certain confidence. To this end, we computed the theoretical decision points that would derive from applying a predefined statistical criterion (here, *p* < 0.05 and *p* < 0.01) to the probability of observing *m* or more sequence element matches between a given stream and the reference stream in an *n*‐long sequence, as derived from the one‐tailed Binomial distribution, with the likelihood of a match for any given sequence element being 0.52 (see Supplementary Methods for details).

As is evident in Figure 4D, the theoretical decision points decrease markedly with increasing probabilistic dependency strengths between the sequences, such that less than 10 elements need to be observed for the fully‐dependent streams (joint probability = 0.4) but more than the total of 50 elements for the only marginally dependent streams (joint probability = 0.2).

Compared to these theoretically derived decision points, the three participant groups’ decision times vary less dramatically as a function of the dependency level and occur markedly later for all but the weakest dependency level. Considering that participants were not explicitly instructed to respond as soon as possible, this likely reflects a general tendency to prioritize accuracy over speed. Supporting this, it is worth noting that performance at the first and final decision points (see Figure S3) was very similar across groups.

## 4 Discussion

We have investigated the impact of early sensory deprivation on a core process of visual processing—the ability to detect temporal dependencies of environmental signals. The main result we report is that, while not fully reaching performance levels of normally sighted controls, late‐sighted patients assessed several years post surgically are able to detect probabilistic dependencies in sensory streams with remarkable above‐chance accuracy. Based on analyses of additional data with longitudinally tracked patients, we discuss the possibility that this skill develops gradually post‐surgery rather than being evident immediately thereafter.

To the extent that such dependency detection is an important strategy for intra‐ and inter‐modal perceptual organization, our results suggest that this mechanism can be acquired, even after many years of early‐onset visual deprivation. Many past studies have pointed to the permanent detrimental impact of early deprivation on dimensions of spatial perception, such as acuity, contrast sensitivity, and face perception (e.g., Kalia et al. 2014; Ganesh et al. 2014; Gupta et al. 2023). Similarly, neuroimaging studies have attested to continued differences in the underlying neuronal organization of the late‐sighted even long after surgery (Hölig et al. 2023; Ossandón et al. 2023). However, as stated at the outset, the pattern of results in the temporal domain appears to be more encouraging (Ellemberg et al. 1999; Ye et al. 2021; Ostrovsky et al. 2009; Orlov et al. 2021). Complementing these past results, we are led to conclude that the resilience of different temporal aspects of sensory information processing to early‐onset, prolonged deprivation is not just confined to low‐level mechanisms but also applies to “higher‐order” aspects of temporal processing, such as the detection of dependencies across extended visual or audio‐visual sequences, enabling the detection of probabilistic dependencies in the sensory environment. The inference we are led to is that temporal processing broadly may not be subject to a strict critical period of acquisition, mitigating its vulnerability to early‐onset, prolonged deprivation, and enabling the detection of probabilistic dependencies in the sensory environment, even if surgical intervention arrives late in the developmental timeline.

A second finding we report is that for the late‐sighted, similar to normally sighted controls, performance in inter‐modal and intra‐modal conditions is comparable. Considering the differences in the development of the visual and the auditory system of a late‐sighted child, this finding attests to their significant plasticity for establishing cross‐modal linkages late in life. It is worth noting that past work has demonstrated that mappings between vision and touch, which constitute a different instance of cross‐modal mappings, in late‐sighted individuals are not established immediately post surgically but develop with experience thereafter (Held et al. 2011; Senna et al. 2021).

A comparison of intra‐ and inter‐modal processing has significance even beyond the domain of development. In the simultaneity detection literature, while some within‐modality assessments have been reported to yield a smaller window of simultaneity than across‐modality comparisons (Mitrani et al. 1986; Dixon and Spitz 1980), other studies have suggested that the two conditions may, in fact, have very similar windows of simultaneity (Levitin et al. 2000). It is unclear how intra‐ and inter‐modal comparisons of simultaneity detection performance would relate to tests of the ability to detect probabilistically dependent sequences in intra and inter‐modal conditions. Additional studies are needed to investigate potential linkages between simultaneity and higher‐order temporal regularity detection.

Finally, we observed that all groups made their decisions later than would be expected when applying a predefined statistical criterion with alpha levels of 0.05 or 0.01. This likely reflects that participants prioritized accuracy over speed and waited until they were very confident before responding. Consistent with this, accuracy at the first and final decision points was nearly identical (see Figure S3). The non‐speeded nature of the task minimized speed‐accuracy tradeoffs and ensured stable estimates of overall performance. Because not all participants in the long‐term follow‐up group provided initial responses, the sample size for analyses of decision times was reduced. However, the lack of an initial response is unlikely to have meaningfully reduced performance, given the uniformly late and highly similar decision patterns across groups. For additional verification, we conducted an online control experiment, where participants completed the same task in both a dual‐response and final‐response‐only mode. Performance did not differ significantly between conditions (paired *t*‐test, *t*(19) = −1.057; *p* = 0.304) and, if anything, was marginally higher in the dual‐response condition (79% vs. 74%) (see Supplementary Methods; Figure S1B). Thus, the requirement to provide an early response does not appear to have reduced performance, and the absence of early responses for part of the long‐term follow‐up group is unlikely to have affected the main conclusions. Nonetheless, the non‐speeded nature of the task reduces the sensitivity of our measure and the interpretability of potential group differences, since all participants adopted a similarly conservative response strategy. Determining the timing of decision‐making in the late‐sighted therefore remains an important goal for future work with speeded paradigms or markedly shorter stimulus sequences.

A comparison of the longitudinal and long‐term follow‐up groups revealed that while children assessed within the first month post‐surgery do not yet exhibit strong dependency detection capabilities, those tested several years post surgically perform markedly better. This pattern is consistent with the interpretation that several months or years of postoperative experience may be required for the robust emergence of this capacity. However, given the challenges inherent in cross‐sectional comparisons, it is important to consider several differences between the two late‐sighted cohorts that may jointly contribute to the observed differences.

First, the longitudinally tracked cohort was notably younger than the long‐term follow‐up group (mean age: 9.8 vs. 17.5 years). Because patients of the long‐term follow‐up group were each assessed three or more years after surgery, an age difference between cohorts is a natural consequence of the study design and was relatively pronounced in the present sample. As perceptual, cognitive, and executive functions can continue to be refined across childhood and adolescence, we cannot fully disentangle effects of chronological age from effects of postoperative visual experience when comparing these cohorts. Cross‐cohort differences must therefore be interpreted cautiously.

Importantly, the two patient cohorts also differed slightly in their average age at surgery, with the longitudinal group receiving surgical intervention earlier than the long‐term follow‐up group (mean ages: 9.8 vs. 12 years). If age at surgery were a primary factor determining later perceptual outcomes, the longitudinal cohort would be expected to exhibit equal or better performance. The fact that the opposite pattern was observed limits alternative interpretations of the group differences. More broadly, this is consistent with prior work showing that age at surgery does not reliably predict postoperative outcomes even for low‐level spatial skills, such as visual acuity, that are highly sensitive to early deprivation and would thus be expected to correlate most strongly with surgical timing (Ganesh et al. 2014).

Second, the role of visual acuity warrants careful consideration. Importantly, acuity at the time of testing does not appear to reduce performance. The task was intentionally designed to minimize high acuity demands by using large, high‐contrast visual stimuli. To empirically assess whether reduced acuity affects performance, we also conducted an online control experiment in which normally sighted adults completed a shortened version of the task without blur as well as with on‐screen blur corresponding to 20/500 vision. Performance did not differ significantly between the no‐blur (Performance mean: 76%) and blurred (Performance mean: 77%) conditions (paired *t*‐test, *t*(19) = −0.145, *p* = 0.886; see Figure S1A and Supplementary Methods). These results indicate that relatively low acuity is sufficient to perform the dependency detection task itself at high levels. This, in part, also helps explain why the longitudinally assessed group demonstrated above‐chance performance even prior to surgery, despite severe visual impairment.

At the same time, acuity may play a critical role in the development of the underlying ability. Severe blur associated with dense congenital cataracts reduces access to distinct, nearby visual entities, which are precisely those that are most likely to exhibit meaningful temporal covariation in the natural environment. With fewer opportunities to observe individuated elements that covary over time, the development of mechanisms for detecting temporal statistical relationships could plausibly be compromised. Thus, the relevant distinction between the longitudinal and long‐term follow‐up cohorts may not lie solely in the duration of postoperative vision, but in the combined effects of duration and quality of available visual input. A child with only one month of relatively low‐acuity postoperative experience would have had substantially fewer opportunities, along both dimensions, to acquire the relevant temporal associations than a child with several years of moderately restored acuity.

Finally, given the rarity of the population, sample sizes were necessarily modest, increasing the potential impact of interindividual variability in perceptual and cognitive function. It is therefore likely that multiple factors jointly contribute to the observed pattern, including the differences observed between the two late‐sighted cohorts. Future work will be valuable for further dissociating these contributions. In addition, experimental extensions using different sequence lengths, speeded response paradigms, and a broader sampling of dependency strengths could help to further elucidate dependency detection capacity and its link to decision‐making strategies.

Notwithstanding some of these challenges and open questions, children in the long‐term follow‐up group robustly demonstrate strong intra‐ and inter‐modal dependency detection despite having experienced extended early‐onset visual deprivation. This indicates a remarkable degree of resilience in acquiring this foundational perceptual ability, which may require an extended period to fully develop. Taken together, the present results help characterize the development of mechanisms for detecting relationships between environmental entities and suggest that sufficient plasticity for acquiring at least part of this ability remains available late into childhood. These findings have important implications for understanding atypical developmental trajectories and for informing rehabilitation prospects following sight‐restoring interventions.

## Author Contributions

**Priti Gupta**: conceptualization, investigation, data analysis, manuscript preparation. **Lukas Vogelsang**: conceptualization, software, data analysis, manuscript preparation. **Marin Vogelsang**: conceptualization, software, data analysis. **Neil Khemani**: conceptualization, data analysis, **Manvi Jain**: investigation. **Naviya Lall**: investigation, manuscript preparation. **Dhun Verma**: investigation.**Chetan Ralekar**: conceptualization, investigation. **Suma Ganesh**: surgical treatment. **Pawan Sinha**: conceptualization, manuscript preparation, funding acquisition.

## Funding

This work is supported by grant R01EY020517 from the National Eye Institute (NIH) to Pawan Sinha. Project Prakash is also supported by the Nick Simons Foundation, the Halis Foundation, and the Sikand Foundation. Priti Gupta is supported by the Fulbright‐Nehru Academic and Professional Excellence Visiting Scholar Program of the United States ‐ India Educational Foundation and the US Department of State. Lukas Vogelsang is supported by a grant from the Simons Foundation International to the Simons Center for the Social Brain at MIT, and Marin Vogelsang is supported by the Japan Society for the Promotion of Science (JSPS) Overseas Research Fellowship and the Yamada Science Foundation.

## Ethics Statement

The study received approval by the Ethics Committees of MIT and Dr. Shroff's Charity Eye Hospital in India.

## Conflicts of Interest

The authors declare no conflicts of interest.

## Supporting information




**Supporting File 1**: desc70204‐sup‐0001‐SuppMat.docx

## Data Availability

Data are available from the lead author upon request.
