# Supplementary material for: The Role of Early Visual Experience in Cross‐Signal Dependency Detection
Source: Dev Sci. 2026 May 5;29:e70204. doi: 10.1111/desc.70204 (PMC13144708; doi:10.1111/desc.70204)
Supplement: Supplementary file 1 — Supporting File 1: desc70204‐sup‐0001‐SuppMat.docx [file DESC-29-e70204-s001.docx]

**Supplementary Material**

The supplementary material accompanying the main manuscript contains:

Supplementary Methods

Supplementary Figures 1-3

Supplementary Tables 1-5

**Supplementary Methods**

***Pre-operative visual assessments***

We conducted assessments for light perception in all four quadrants and used the Freiburg Visual Acuity Test (Bach, 1996) to measure acuity. All patients were within the categories of ‘Profound visual impairment (20/500 - 20/1000)’ or ‘Light perception/projection (<20/1000)’, in accordance with the classification norms by the American Foundation for the Blind. We evaluated the anterior segment using a slit lamp, and the cataract type and any associated ocular pathology were recorded. The patients’ dense bilateral cataracts precluded fundus viewing via ophthalmoscopes. We therefore recorded B-scan ultrasound to detect major posterior segment pathology pre-operatively.

***Surgical intervention***

For all children, keratometry and biometry were carried out under general anesthesia, just before the surgical procedure. All surgical interventions were performed by a single surgeon. As part of the surgeries, the patients underwent a primary posterior capsulorhexis through the anterior route, with a foldable acrylic posterior chamber intra-ocular lens (PCIOL) implanted in the bag. Patients were prescribed refractive correction after suture removal.

***Comparison of decision time with a pre-defined statistical criterion***We examined the amount of information theoretically required to arrive at a decision regarding the more dependent sensory stream. We did so with the following rationale: If one were to determine whether a given sensory stream is probabilistically linked with the reference stream, then every time a newly appended sequence element (beginning with element 1, all the way to element 50) is identical to the one appended to the reference stream, it constitutes a match success for the corresponding stream. Streams that are independent from the reference will show, on average over time, 52% matches (not 50%, as the prior probabilities of ‘0’ and ‘1’ are 0.6 and 0.4, yielding a 0.6*0.6 + 0.4*0.4 = 0.52 chance-level match), while a dependent stream will have a higher proportion of matches.

The probability of observing *m* or more matches in an *n*-long sequence can be derived from the one-tailed Binomial distribution, with the likelihood of a match for any given sequence element being 0.52. Hence, for each sequence, we can compute a running sequence of p-values based on the number of matches observed thus far. P-values for streams that are independent from the reference will likely not reach significance, while those associated with a dependent stream will show a progressive reduction with increasing length of the sequence observed, eventually attaining some criterion of statistical significance. At this point, a hypothetical statistician will be able to select, with a high degree of confidence, the stream most strongly linked with the reference. (For example, if over the course of 20 elements, a particular stream exhibits 15 or more matches, the probability of that happening by chance is approximately 0.02. Given this low likelihood, it is reasonable to infer that this stream is likely associated with the reference.) The higher the probabilistic dependency between one of the sensory streams and the reference stream, the sooner this decision point would be expected to be reached. Given the decision point corresponding to a defined level of statistical significance (here, we chose 0.05 and 0.01), we can examine how it compares with that of a human observer.

***Online validation experiments***

We conducted two online validation experiments with normally sighted adults to assess whether (i) the dual-response instruction and (ii) visual blur influenced task performance. These experiments were designed solely to validate task characteristics in a within-subjects manner and were not intended as tests of group differences or any developmental effects. Participants were recruited via Prolific and were between 18 and 22 years of age. Recruitment was not intended to match the socioeconomic background of the patient groups.

In the first online experiment, we examined whether blur (corresponding to approximately 20/500 acuity) affected performance on the dependency detection task. Twenty participants each completed 10 trials at the most challenging dependency level tested in our main study (joint probability = 0.2). The dependency level was selected to minimize the chance of ceiling effects in the online sample. For each participant, five trials were presented with no blur and five with simulated blur corresponding approximately to 20/500 vision. Trials were presented in a randomly shuffled order. The stimulus sequences were identical to those used in the main in-lab experiment. As shown in Supplementary Fig. 1A, this experiment revealed that performance did not differ significantly between blurred and non-blurred conditions, indicating that performance is robust to reductions in visual acuity.

A separate group of 20 participants completed a second online experiment conducted to assess whether the task requirement to provide both an early and a final response affects performance. Participants completed 10 trials when instructed to provide only a final response, and 10 trials when instructed to provide both an initial and a final response. Each condition included five trials at each of the two most difficult dependency levels (joint probabilities of 0.2 and 0.28), with trial order counterbalanced across participants. As shown in Supplementary Fig. 1B, no reliable performance difference was observed between the two instruction conditions. Thus, the requirement to provide an early response did not systematically affect performance.

Together, these validation experiments confirm that neither reduced spatial resolution nor the dual-response instruction strongly influenced performance in the dependency detection task.

**Supplementary Figures**

*
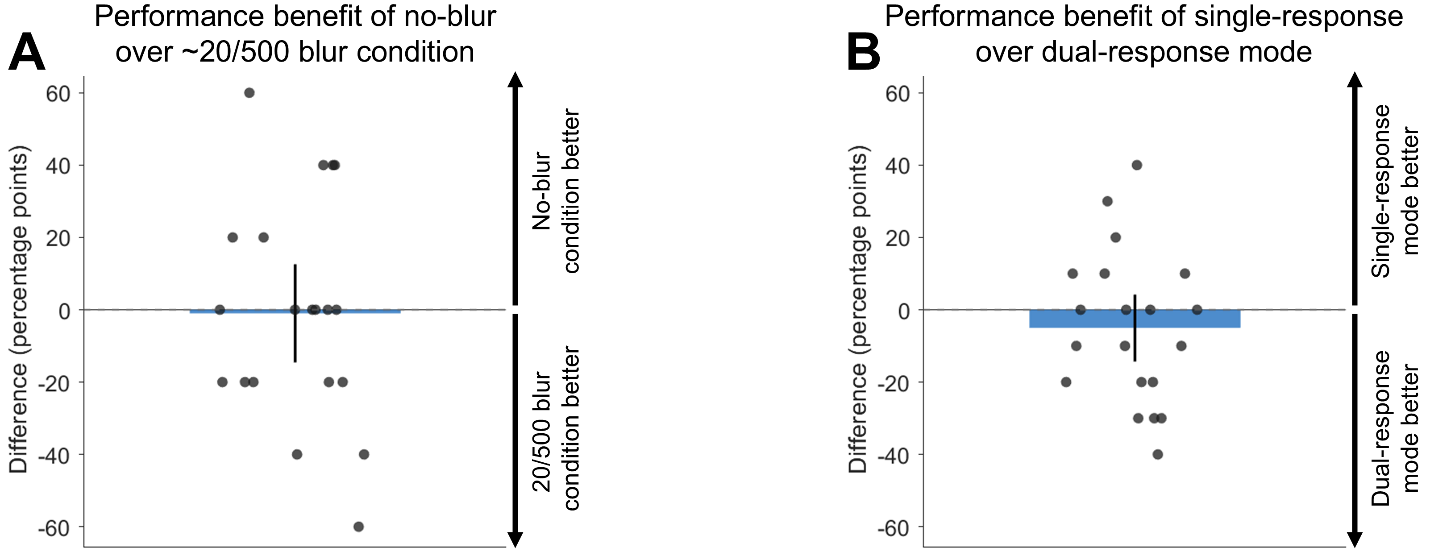
*

***Supplementary Fig. 1.*** *Two additional online experiments conducted with 20 participants each.* ***A.*** *Performance difference (in percentage points) when completing the task without visual blur vs. with blur corresponding to 20/500 vision. No significant difference was found (paired t-test, t(19) = -0.145, p = 0.886).* ***B.*** *Performance difference (in percentage points) when completing the task in a single-response mode (only providing a final response) vs. in a dual-response mode (providing both an initial and a final response). No significant difference was found (paired t-test, t(19) = -1.057; p = 0.304).*

*
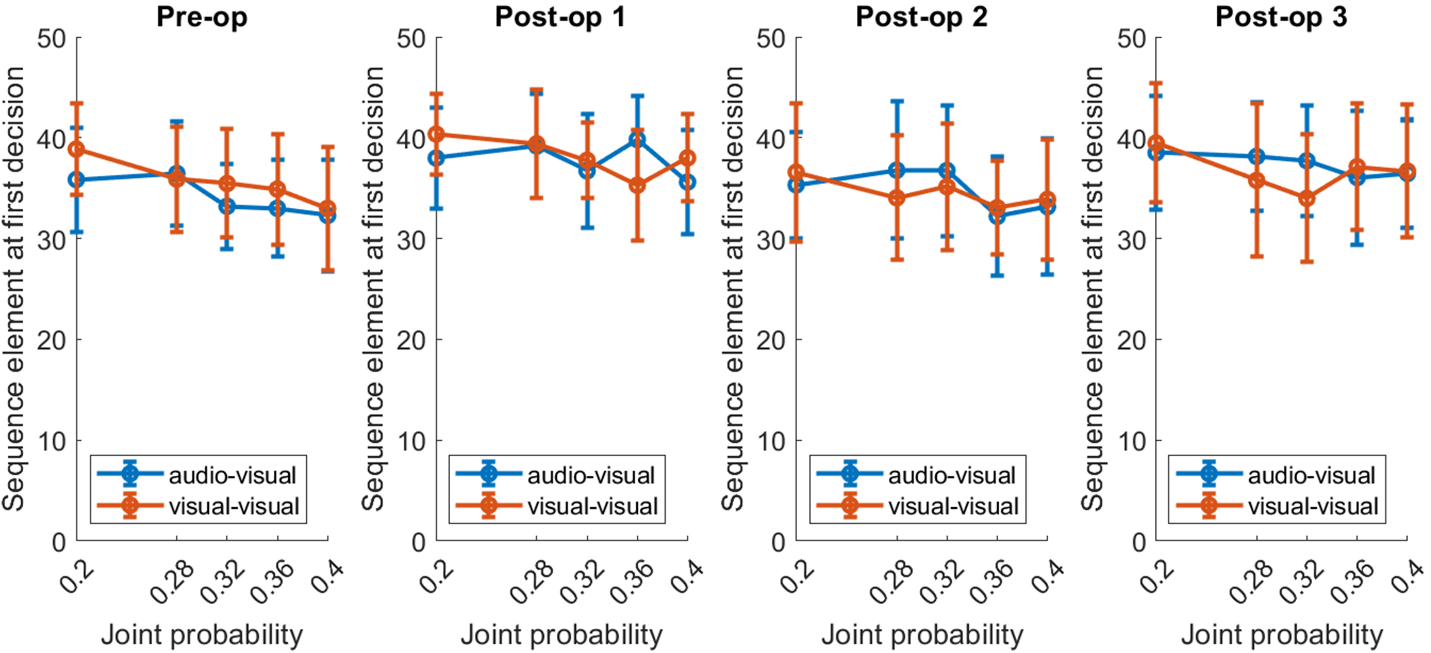
*

***Supplementary Fig. 2.*** *Sequence element at which the first decision was made, depicted for the longitudinal group (n=12) at the pre-operative and the three post-operative time points (1 week, 2 weeks, and 1-month post-surgery), as a function of dependency strength (0.2 = weakly-dependent (0.16 would correspond to fully-independent); 0.4 = fully-dependent) and modality (audio-visual vs. visual-visual).*

*
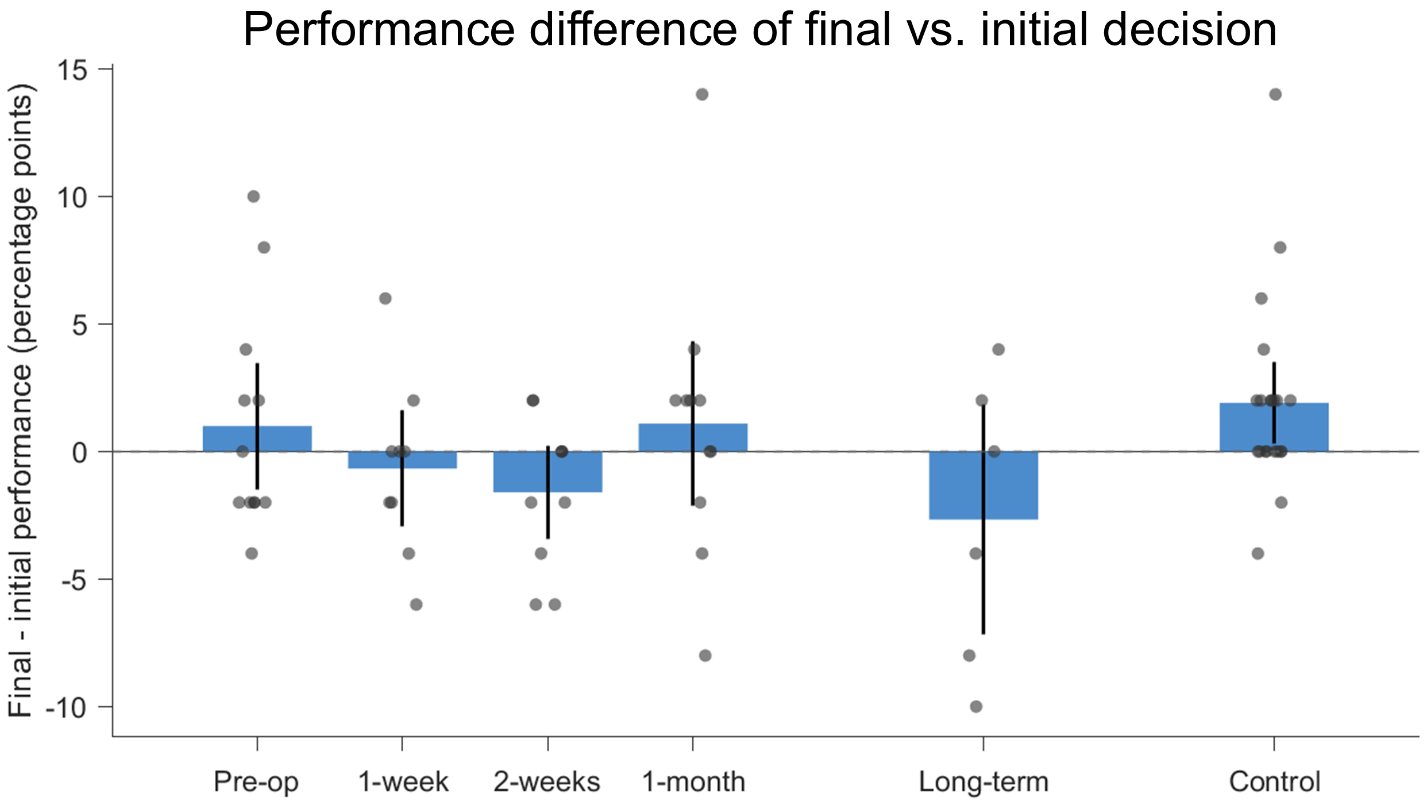
*

***Supplementary Fig. 3.*** *Performance difference (in percentage points) at the initial vs. final decision point, depicted for the longitudinally-tracked group at four different timepoints, the long-term late-sighted group, and the control group. Error bars depict 95% confidence intervals.*

**Supplementary Tables**

| **Participant** | **Age** | **Gender** | **Age at treatment** | **Pre-treatment acuity (Snellen)** | **Post-treatment acuity (Snellen)** | **Years since surgery** |
| --- | --- | --- | --- | --- | --- | --- |
| Patient 1 | 11 | F | 8 | 20/9267 | 20/182 | 3 |
|  |  |  |  |  |  |  |
| Patient 2 | 13 | F | 10 | 20/541 | 20/191 | 4 |
| Patient 3 | 19 | M | 15 | 20/1617 | 20/575 | 4 |
| Patient 4 | 22 | M | 10 | Could count fingers at 1m | 20/380 | 12 |
| Patient 5 | 20 | M | 16 | 20/969 | 20/207 | 4 |
| Patient 6 | 24 | M | 14 | Could only perceive hand movements close to face | 20/265 | 10 |
| Patient 7 | 22 | M | 13 | Could count fingers at 0.5m | 20/241 | 9 |
| Patient 8 | 15 | F | 12 | 20/9267 | 20/1782 | 3 |
|  |  |  |  |  |  |  |
| Patient 9 | 17 | F | 12 | 20/627 | 20/231 | 4 |
| Patient 10 | 22 | M | 12 | Could only perceive hand movements close to face | 20/2276 | 10 |
| Patient 11 | 15 | M | 11 | 20/2592 | 20/235 | 4 |
| Patient 12 | 17 | F | 13 | 20/853 | 20/195 | 4 |
| Patient 13 | 15 | M | 11 | 20/1251 | 20/387 | 4 |
| Patient 14 | 17 | M | 13 | 20/751 | 20/162 | 4 |
| Patient 15 | 14 | M | 10 | 20/839 | 20/182 | 4 |

***Supplementary Table 1.*** *Detailed information of long-term follow-up late-sighted patients. Visual acuity values are reported with best visual correction.*

| **Participant** | **Age** | **Gender** | **Pre-treatment acuity (Snellen)** | **Post-treatment acuity (Snellen)** |
| --- | --- | --- | --- | --- |
| Patient 1 | 17 | M | 20/1725 | 20/1538 |
| Patient 2 | 7 | F | 20/692 | 20/370 |
| Patient 3 | 11 | F | 20/567 | 20/429 |
| Patient 4 | 8 | F | 20/1297 | 20/456 |
| Patient 5 | 9 | F | 20/3474 | 20/1622 |
| Patient 6 | 13 | F | 20/781 | 20/203 |
| Patient 7 | 11 | M | 20/1085 | 20/191 |
| Patient 8 | 4 | F | 20/1769 | 20/893 |
| Patient 9 | 6 | M | 20/406 | 20/399 |
| Patient 10 | 9 | F | 20/708 | 20/447 |
| Patient 11 | 15 | F | 20/559 | 20/262 |
| Patient 12 | 7 | F | 20/634 | 20/499 |

***Supplementary Table 2.*** *Detailed information of longitudinally-tracked late-sighted patients. Visual acuity values are reported with best visual correction.*

| **Participant** | **Age** | **Gender** |
| --- | --- | --- |
| Participant 1 | 18 | F |
| Participant 2 | 18 | F |
| Participant 3 | 17 | F |
| Participant 4 | 21 | F |
| Participant 5 | 20 | F |
| Participant 6 | 19 | F |
| Participant 7 | 18 | F |
| Participant 8 | 19 | F |
| Participant 9 | 24 | F |
| Participant 10 | 18 | F |
| Participant 11 | 21 | F |
| Participant 12 | 18 | F |
| Participant 13 | 18 | F |
| Participant 14 | 18 | F |
| Participant 15 | 17 | F |
| Participant 16 | 22 | F |
| Participant 17 | 18 | F |
| Participant 18 | 18 | F |
| Participant 19 | 18 | F |
| Participant 20 | 19 | F |
| Participant 21 | 17 | F |

***Supplementary Table 3.*** *Age and gender of individual participants of the control group.*

| **Probability** | **Modality** | **P value** |
| --- | --- | --- |
| 0.2 | Audio-visual | 0.910 |
| 0.2 | Visual-visual | 0.485 |
| 0.28 | Audio-visual | 0.001* |
| 0.28 | Visual-visual | <0.001* |
| 0.32 | Audio-visual | 0.004* |
| 0.32 | Visual-visual | <0.001* |
| 0.36 | Audio-visual | <0.001* |
| 0.36 | Visual-visual | <0.001* |
| 0.4 | Audio-visual | <0.001* |
| 0.4 | Visual-visual | <0.001* |

***Supplementary Table 4.*** *Results of one-sample Wilcoxon signed-rank tests comparing performance of the long-term late-sighted patient group, for each probability / modality combination, with chance level performance (50% correct responses). An asterisk indicates statistical significance following Bonferroni correction (p < 0.05/10).*

| **Probability** | **Modality** | **P value** |
| --- | --- | --- |
| 0.2 | Audio-visual | <0.001* |
| 0.2 | Visual-visual | <0.001* |
| 0.28 | Audio-visual | <0.001* |
| 0.28 | Visual-visual | <0.001* |
| 0.32 | Audio-visual | <0.001* |
| 0.32 | Visual-visual | <0.001* |
| 0.36 | Audio-visual | <0.001* |
| 0.36 | Visual-visual | <0.001* |
| 0.4 | Audio-visual | <0.001* |
| 0.4 | Visual-visual | <0.001* |

**Supplementary Table 5.** Results of one-sample Wilcoxon signed-rank tests comparing performance of the control group, for each probability / modality combination, with chance level performance (50% correct responses). An asterisk indicates statistical significance following Bonferroni correction (p < 0.05/10).
